# Apps in Clinical Practice: Usage Behaviour of Trauma Surgeons and Radiologists in Northern Germany

**DOI:** 10.1155/2023/3930820

**Published:** 2023-08-02

**Authors:** Marie Keitsch, Alonja Reiter, André Strahl, Karl-Heinz Frosch, Josephine Berger-Groch, Matthias Priemel

**Affiliations:** ^1^Department of Trauma and Orthopeadic Surgery, University Medical Center Hamburg-Eppendorf, Martinistr. 52 20246 Hamburg, Germany; ^2^Department of Trauma Surgery and Orthopeadics, Pediatric Orthopeadics, Agaplesion Diakonieklinikum Rotenburg, Elise-Averdieck-Str. 17, 27356 Rotenburg (Wümme), Germany; ^3^Department of Trauma Surgery, Orthopeadics and Sports Traumatology, BG Hospital Hamburg, Bergedorfer Str. 10, 21033 Hamburg, Germany; ^4^Department of Trauma and Orthopeadic Surgery, Katharinenhospital Klinikum Stuttgart, Kriegsbergstr. 60 70714 Stuttgart, Germany

## Abstract

**Introduction:**

Apps, in general, are an integral part of our daily lives. To investigate the current usage behaviour of trauma surgeons and radiologists regarding medical apps in clinical practice and to find out if and how the current range of medical apps can be improved, we surveyed trauma surgeons and radiologists in northern Germany. *Material and Methods*. An online questionnaire was sent to 100 trauma surgeons and 100 radiologists in northern Germany. Participants were asked about the frequency of their use of medical apps in clinical practice, which apps were used most often, how useful participants thought apps were, and in which area they would like to see improvements. The most frequently mentioned apps were finally analyzed.

**Results:**

The survey study showed that 87.4% of the trauma surgeons and 67.4% of the radiologists use medical apps on a regular basis at work. It also revealed that trauma surgeons used medical apps much more often than radiologists and that young doctors were more likely to rely on medical apps than chief physicians. 80.0% of the participants would pay at least 5 euros for a medical app. Trauma surgeons see the greatest need for support in their daily work from medical apps in the area of treatment, while radiologists seek more support in the area of classification.

**Conclusion:**

The study underscored the broad acceptance of medical apps in everyday clinical practice. As the physicians are willing to spend money and stated a general interest and need for further developments, there is high potential for the future. This trial is registered with DRKS00026766.

## 1. Introduction

Not only have smartphones become omnipresent in our day-to-day living but are also an integral part of clinical practice and medical education. Due to the increasing penetration of mobile devices and low mobile prices, the share of mobile Internet users in Germany has steadily increased in recent years and amounted to 80% in 2020, while in 2015, the share of mobile Internet users was still 54% [1]. The number of smartphone users in Germany is 60.7 million in 2021, and it is estimated to increase to 68.6 million by 2023 [2]. In November 2021, there were 3,325,891 apps available in the Google Play Store and 1,862,236 apps available in the iOS App Store. Hundreds to thousands of new apps appear on the Google Play Store and iOS App Store, respectively, every day [3]. Only 3.5% were from the “health and fitness” category, and 1.93% were from the “medical” category [4].

In recent years, physical medical treatment and education have been partially replaced by digital technologies such as telemedicine and virtual services practices [5, 6]. Accordingly, the prevalence of telemedicine and virtual treatment has increased rapidly [7, 8], and health care organizations have adopted digital solutions and advanced technology tools such as artificial intelligence- (AI-) based diagnostic algorithms based both on imaging and clinical data [9, 10]. In other areas, too, other ways were introduced. For example, new apps were invented to move the fellowship and residency interviews to a virtual format [11, 12].

Since the introduction of the smartphone in 2007, it has become an indispensable part of a doctor's everyday work: For communication with colleagues [13–15], as a classification or calculation aid [16–18], or simply as a reference tool. The smartphone has already almost completely replaced the classic look into the textbook, especially among younger colleagues [19, 20].

While the treatment of musculoskeletal tumours should be performed in specialized centres due to the complexity of these diseases, the initial diagnosis of musculoskeletal tumours is a common challenge for non-specialized physicians—trauma surgeons and radiologists in particular—in facilities at all levels of care. Especially for rare diseases, the support of physicians by medical applications could be helpful [21]. Unfortunately, the range of helpful apps in this area of medicine is still very scarce [22].

The aim of the survey was to find out the current status of the use of medical apps in everyday clinical practice in the specialties of trauma surgery and radiology to determine if there is a need to improve the current app offerings, especially when it comes to diagnosing musculoskeletal tumours.

## 2. Material and Methods

Based on a systematic review by our research group [22], which found that no app is yet available to actively assist physicians in diagnosing musculoskeletal tumors, an online survey was conducted among trauma surgeons and radiologists for market analysis. The research group team identified questions for this survey. For this purpose, factors relevant to the topic were narrowed down according to the existing literature [23, 24]. We formulated questions with which the medical profession as a whole can be divided into different groups in order to better analyze the usage behavior of those groups—for example, age, level of continuing education—so that the corresponding target groups can be better addressed in later projects. Then a digital questionnaire was created. The survey was sent to trauma surgeons and radiologists in northern Germany. The inclusion criteria for participants were (1) completed medical studies, (2) employment in northern Germany, and (3) at least 6 of the 10 questions were answered. Using the Clopper-Pearson confidence interval calculation, a target number of 100 participants per group was established. Chief physicians of hospitals in northern Germany were contacted directly via the email addresses of the clinic homepage. Physicians in private practice were contacted via associations of the professional bodies of radiology and trauma surgery. The survey was created using the free online survey tool “easyfeedback” [25]. When creating the survey, a link was generated, which was then sent to the participants. Via the link provided in the email, the participants were directly taken to the digital survey without having to register or enter any personal data. Participation was completely anonymous. Only the account holder was able to view the survey results, so data protection was ensured. Two separate survey links were generated for surgeons and radiologists. The content of the two surveys was identical. The surveys consisted of 10 questions. The questions were both closed and open-ended. The questions could be answered by the participants without time pressure. Subsequent changes to the answers were also possible by going back in the survey, and multiple answers were possible. The survey started on 04/01/2021 and was closed on 12/17/2021, when 100 physicians in each group had participated.

### 2.1. Survey Subsections

Participants were asked about their age, gender, and level of training/rank, how often they used medical apps in their daily clinical practice, whether they named the 5 apps they used most often, and whether they considered apps useful in general. Furthermore, participants were asked which operating system was used, whether they would invest money in medical apps, and in which area they would like to see improvement in medical apps. In terms of further work, participants were also asked how they would behave when confronted with a musculoskeletal tumour on X-ray. Multiple responses were possible for the last two questions. The full survey can be seen in supplement 1.

### 2.2. Statistical Analysis

Survey results were transferred to an Excel spreadsheet (Excel version 2016, Microsoft Corp., Redmond, WA, USA), and descriptive statistics were calculated for all subitems. A separate Internet search was performed for the apps most frequently mentioned by trauma surgeons and radiologists, and the apps were ranked and evaluated with respect to category, creation date/last update, origin, price, rating, and user. Descriptive statistics were used to describe the basic characteristics of the data set. Continuous variables were expressed as mean and standard deviation (SD), while categorical variables were expressed as number and percentage. Differences between groups were calculated using the Mann–Whitney *U* test for nonnormally distributed data or the Chi^2^-test. If several independent variables were to be compared, the Kruskal-Wallis test was applied. A *p* value <0.05 was considered statistically significant. Statistical analyses were performed using a SPSS statistical software (SPSS version 27.0, Chicago, IL, USA).

## 3. Results

In both groups, 95/100 participants were included in the evaluation. Surveys in which a participant answered only 5 or less questions were considered incomplete. Among both trauma surgeons and radiologists, 5 individuals completed the survey incompletely, so they were excluded.  Table 1 shows the distribution of gender, different age groups, and in different levels of training. No significant differences were found within these variables (*p* > 0.05).

The study showed clear differences in the usage patterns among the different education levels (*p*_Kruskal−Wallis−H_ = 0.02). Among the surgeons´ residents, 35 out of the 38 participants (92.1%) use medical apps regularly—at least once a week or more frequently. Three participants (7.9%) use them only once per month or never. In the group of the senior and chief physicians only 66.7% and 57.1%, respectively, use medical apps regularly, whereas 33.3% and 42.9%, respectively, seldom or never use them. See Table 2.

Our analysis demonstrated as well that the variety of apps mentioned by the different education levels differs greatly. While the surgeons' residents listed 127 different apps, the senior physicians named only 65 different apps, and the chief physicians with only 7 different apps even less (*p*_Chi_^2^ < 0.001). The distribution was similar among radiologists (*p*_Chi_^2^ < 0.001) and in the overall sample (*p*_Chi_^2^ < 0.001).

68.9% of the participants used the iOS operating system, and 31.1% used the android operating system (*p* < 0.001). No other operating systems such as Windows Phone or Blackberry were mentioned.

With a total of 147 out of the 190 participants (77.4%), the majority described medical apps as helpful and used them regularly during their daily work. Looking at the two medical specialties separately, it was 67.4% (64 participants) among the radiologists and 87.4% (83 participants) among the trauma surgeons. Overall, 43 participants (22.6%) did not use them regularly. Here, the distribution was 12.6% among the trauma surgeons and 32.6% among the radiologists. See Table 3. Overall, trauma surgeons considered the use of apps to be statistically more helpful than radiologists (*p*_*U*−Test_ = 0.001).

15.8% of all respondents were not willing to spend money on medical apps, while 80.0% would pay at least 5 euros, and about one third (30.0%) would pay more than 10 euros per app. See Table 3.

Among trauma surgeons, only 9 participants (9.5%) reported having no medical apps on their smartphone, whereas the number among radiologists was much higher with 33 participants (34.7%) (*p*_Chi_^2^ = 0.001). Among trauma surgeons, 77 different apps were listed, and among radiologists, 60 different apps were listed. Those apps with usage rates above 10% are assembled in Table 4.

The full app ranking of both trauma surgeons and radiologists is displayed in supplements 2 and 3. In both groups, the majority of apps were mentioned in the category “reference” (among the trauma surgeons, 23 apps and among radiologists, 17 apps). In the surgeons' group, the remaining distribution was as follows: 12 apps in the category “treatment,” 9 apps in the category “calculator,” 5 apps each in the categories “education” and “medication,“ and 3 apps in the category “classification.” Overall, only one app called mRay was mentioned in the category “imaging” by the trauma surgeons (3 mentions). Among the radiologists, on the other hand, there were 11 apps listed in the category “imaging,” 7 apps in the category “calculator,” 4 apps in the category “education,” 3 apps in the category “medication,” and two apps each in the categories “treatment” and “communication”.

With the survey, we also wanted to determine in which areas participants would like to see more support from medical apps. In the trauma surgeon group, treatment was the most frequently mentioned area, followed closely by imaging and classification, whereas the radiologists hardly indicated any further support in the area of treatment but great need in the area of classification. See Table 5.

The last question was about how participants would proceed if they diagnosed a musculoskeletal tumour. The majority in both groups stated that they would inform themselves on the Internet. The differences between the two specialties were primarily that the surgeons would consult a reference book or refer the patient to a specialty centre in equal shares, whereas the radiologists would clearly prefer a reference book over a specialty centre. The exact numbers are shown in Table 6.

## 4. Discussion

With our survey study, we were able to show that medical apps are being used by physicians in everyday clinical practice more frequently than ever before. The use of smartphones and apps among physicians has already been investigated in several studies. In 2007, a Europe-wide study on the use of smartphones and apps in the specialty of trauma surgery found that 73.6% of the respondents had medical apps on their smartphones, but 60.0% never or rarely used them, whereas nonmedical apps such as Messenger and WhatsApp or the integrated camera for clinical documentation were used by 75.9% of the respondents. Only 24.1% described the available medical apps as useful for use in clinical practice [26]. In 2010, a survey of orthopaedic surgeons in the United Kingdom found out that 84% of respondents owned a smartphone, but only 53% used medical apps in clinical practice [27]. In 2012, a multidisciplinary survey of physicians from 27 different specialties found out that 56% used medical apps in their daily work [28]. And most recently, in 2018/2019, a survey conducted among trauma surgeons across Germany on the use of medical apps in everyday clinical practice showed that apps were regularly used by 64.7% of the respondents in their daily work and research [24]. Thus, a steady increase in usage has been observed, and our study has shown that the trend is continuing. More so, with now 77.4% in the two specialties investigated combined and 87.4% in the trauma surgeon group in particular, we could detect a clear increase. This suggests that demand in this area has not yet been met. On the contrary, this sharp increase in usage shows that digitization in the field of trauma surgery holds enormous potential for the development of further mobile tools.

This is further reinforced by the question about what to do in the event of a musculoskeletal tumor, where the majority of respondents said they would seek help online, indicating a need for more support. See Table 6. A systematic review performed by our research group revealed that nearly all medical applications currently available in the Apple App Store or the Google Play Store are concerned with conveying learning content or imparting knowledge [22]. So far, no application is available that actively supports the physician in finding a diagnosis or suggests therapeutic options for a specific case. The potential for new apps to be developed is therefore great.

Another finding of our study was that the most frequently used apps (see Table 3) all have professional background and were developed especially for doctors, medical students, and/or medical staff, which could be a plausible explanation for why these apps have been able to establish themselves repeatedly. This shows that in order to make a selection from the multitude of apps on offer, it is essential for the physician to be able to assess: How valid is the app, and can it be relied upon for patient care? Therefore, independent recurring evaluations are important from a scientific perspective. The range of available apps in trauma surgery has been reviewed in the past. In 2015, 76 apps were identified, of which 45 (59%) were claimed to be medical apps and 28 (37%) were so-called “health and fitness” apps. But only 30 of these apps (39%) had professional medical involvement in their development and content [29]. Another study in 2019 investigated which apps were mainly used by trauma surgeons in Germany and identified 13 relevant apps [23]. All of the most frequently mentioned apps from our study in the surgeons' group match that list, and two apps (Amboss and Arznei aktuell) were also part of the radiologists' ranking. To see a detailed description of the most mentioned apps from our study, see supplement 4.

Furthermore, the present study showed that with 87.4% vs. 67.4% trauma surgeons were much more likely to use apps than radiologists. The reason for this could be that radiologists do most of their work on a computer and use the Internet browser on their PC when they have questions or are uncertain, rather than picking up a second digital device. Trauma surgeons are more mobile in their daily work due to rounds and consultations and are therefore more likely to use a mobile device for research. It also became apparent that the two specialties have different wishes with regard to new apps. For surgeons, the focus is mainly on treatment, while this area plays a rather subordinate role for radiologists, who would like to see more support from apps in the area of classifications. This should be taken into account in the development of new apps.

We could also show that residents use more different apps and those apps more frequently than senior and chief physicians. This could be due to the fact that the younger generation of physicians came into contact with digital media much earlier in life, so-called “early adopters,” and naturally learned to integrate smartphones and apps into their daily lives. According to a study from 2012, medical students use smartphone and medical apps for learning [19], while junior doctors often use smartphone apps that provide hospital-specific guidelines and management algorithms for common conditions which are useful on busy wards with limited number of computers and provide rapid access to information in high-pressure situations [20]. And since the number of patients to be treated is increasing—both on the wards, where there is more fluctuation as the patients' length of stay is kept shorter and shorter due to the requirements given by the Medical Service of the Health Insurance (German: Medizinischer Dienst der Krankenkassen, MDK), and in the consultation hours, where more and more patients have to be treated in an ever shorter time—it is not surprising that especially younger doctors are relying more and more on digital media in which classifications, medication dosages, or even treatment paths can be read quickly and reliably. Thus, the target group for new developments is more likely the age group between 24 and 34, as this group has a significantly higher interest in apps.

The study also showed that the majority of physicians are willing to invest money in a medical app. Also, only 10.0% of the participants in our study stated that they did not need any more apps to support them during their daily work, whereas more than half of the participants would like to have more support by medical apps in at least 3 different categories. This shows that the need for reliable sources is great. Among the multitude of apps touted as medical apps or mHealth apps in the App Store, there are comparatively few that can be used reliably—legally and ethically adequately—by physicians [23]. An example of this is the app WhatsApp, which is used by many colleagues for communication [30], but in doing so does not meet the specifications of data security and in principle even violates medical confidentiality [31]. The Siilo app, on the other hand, is now a data-secure app especially developed for medical messaging that is gaining ground. In our study, this app was mentioned in both participation groups (see supplement 2 and 3) but with a usage rate of only 8.4% among the surgeons and 4.7% among the radiologists, it did not make the top 5 lists—probably due to the fact that the app is comparatively young.

## 5. Limitations of the Work

Since the number of participants in the survey was limited, the trauma surgeons and radiologists that participated in the survey may not be representative of the entirety of trauma surgeons and radiologists in Germany. It can also be assumed that primarily smartphone-savvy trauma surgeons and radiologists participated in our survey, which distorts the smartphone usage rate. Also, due to the study design, no reliable statement can be made about the response rate since several potential study participants could be reached with one sent invitation email.

## 6. Conclusion

Our study underscores the wide acceptance of medical apps in everyday clinical practice in both specialties, but especially among trauma surgeons, who are significantly more likely than radiologists to use apps in their work routine. More so, it is an undeniable fact that the use of smartphones in clinical practice is an established reality. Physicians regularly resort to medical apps, especially when they have professional background and they are willing to pay for a reliable app. This suggests that digitization—especially in the field of trauma surgery—holds enormous potential for the development of further mobile tools. It was also shown that the different specialties have different needs regarding professional support, which should be considered when developing new apps. In the field of traumatology, new apps that specifically address the treatment of injuries or diseases would meet the current need the most, according to our study, followed by apps that assist physicians in fracture classification and those that offer assistance in interpreting X-ray, CT, or MRI images. In the field of radiology, the needs are understandably situated differently. Radiology colleagues work largely in diagnostics, so apps that focus on injury/disease management are not much needed here, whereas participants in our study would like more support in classification and imaging. Thus, future apps that focus on these two areas are likely to be readily adopted. Also, the young generation in doctors is more likely to rely on medical apps than chief physicians. A finding that coincides with the results of other studies [20, 24]. Consequently, when developing new apps, it should be kept in mind that the design tends to be geared towards the younger generation of the medical profession.

## Figures and Tables

**Table 1:** Gender, age, and hierarchy distribution in comparison of trauma surgeons vs. radiologists.

	Trauma surgeons	Radiologists
*Gender*		
Female	45 (47.4%)	40 (42.1%)
Male	50 (52.6%)	54 (56.8%)
*Age*		
24-34 years	37 (38.9%)	31 (32.6%)
35-45 years	45 (47.4%)	34 (35.8%)
46-56 years	8 (8.4%)	20 (21.1%)
57-67 years	5 (5.3%)	8 (8.4%)
*Education level*		
Residents	38 (40.0%)	29 (30.5%)
Senior physician	27 (28.4%)	24 (25.3%)
Medical specialist	17 (17.9%)	19 (20.0%)
Chief medical officer	7 (7.4%)	7 (7.4%)
Doctor in private practice	5 (5.3%)	15 (15.8%)

**Table 2:** Usage pattern among the different levels of education in trauma surgeons.

	Several times/day	1x/day	1x/week	1x/month	Never
Residents (*n* = 38)	3	20	12	1	2
Senior physician (*n* = 27)	2	4	12	6	3
Chief medical officer (*n* = 7)	0	1	3	0	3
Medical specialist (*n* = 17)	3	4	4	4	2
Doctor in private practice (*n* = 5)	0	1	0	2	2

**Table 3:** Participants' assessment of the usefulness of medical apps and investment.

	Trauma surgeons	Radiologists	p-value
*Usefulness*			
Yes, no longer imaginable without	25 (26.3%)	14 (15.8%)	0.001
Yes, partially	58 (61.1%)	50 (52.6%)	
No, too complicated	1 (1.1%)	11 (11.6%)	
No, I do not know what the possibilities are	7 (7.4%)	17 (17.9%)	
No, does not help me in the end	4 (4.2%)	3 (3.2%)	
*Investment*			
Nothing	15 (15.8%)	15 (15.8%)	0.431
Up to 1 euro	3 (3.2%)	2 (2.1%)	
Up to 5 euro	36 (37.9%)	25 (26.3%)	
Up to 10 euro	12 (12.6%)	22 (23.2%)	
More than 10 euro	29 (30.5%)	28 (29.5%)	

**Table 4:** Top 5 apps of trauma surgeons and radiologists with corresponding usage rates.

Trauma surgeons (*n* = 77)	Usage rate	Radiologists (*n* = 60)	Usage rate
AO surgery reference	39.8%	MRI-Essentials	26.6%
Arznei aktuell	39.8%	IMAIOS e-Anatomy	26.6%
Amboss	27.7%	eRef Thieme	26.6%
Orthora	16.9%	Amboss	20.3%
AO classification	15.7%	Arznei aktuell	12.5%

**Table 5:** More support in everyday work through medical apps.

	Trauma surgeons	Radiologists
Classification area	52 (54.7%)	72(75.8%)
Imaging area	51 (53.7%)	56 (58.9%)
Treatment area	61 (64.2%)	18 (18.9%)
Education area	43 (45.3%)	39 (41.1%)
No more apps	8 (8.4%)	11 (11.6%)

**Table 6:** Procedure in the case of a tumour in the musculoskeletal system.

	Trauma surgeons	Radiologists
Reference book	44 (46.3%)	64 (67.4%)
Internet	62 (65.3%)	77 (81.1%)
Colleague	53 (55.8%)	51 (53.7%)
Specialty center	40 (42.1%)	11 (11.6%)
No help needed	2 (2.1%)	12 (12.6%)

## Data Availability

The detailed survey results used to support the findings of this study are listed in an Excel spreadsheet and are available from the corresponding author upon request.

## Supplementary Materials

Supplementary 1 The survey.

Supplementary 2 App ranking of the trauma surgeons.

Supplementary 3 App ranking of the radiologists.

Supplementary 4 Description of the most mentioned apps.
