# Supplementary material for: Apps in Clinical Practice: Usage Behaviour of Trauma Surgeons and Radiologists in Northern Germany
Source: Int J Telemed Appl. 2023 Aug 2;2023:3930820. doi: 10.1155/2023/3930820 (PMC10412380; doi:10.1155/2023/3930820)
Supplement: Supplementary 1 — The survey. [file 3930820.f1.docx]

**The survey**

**Question 1**

Are you

- female
- male
- diverse

**Question 2**

What age group are you in

- under 24 years
- 24-24 years
- 35-45 years
- 46-56 years
- 57-67 years

**Question 3**

Are you

- Resident
- Medical specialist
- Senior physician
- Chief medical officer
- Doctor in a private practice

**Question 4**

What medical apps do you have installed on your smartphone?

(name the apps, if you do not have any apps write „none“)

__________________________________________

__________________________________________

__________________________________________

__________________________________________

__________________________________________

**Question 5**

How often do you use medical apps during your daily work routine

- never
- 1x per month
- 1x per week
- 1x daily
- Several times a day

**Question 6**

Do you consider medical apps as useful?

- Yes, no longer imaginable without
- Yes, partially
- No, too complicated
- No, does not help me in the end
- No, I do not know what the possibilities are

**Question 7**

What operating system do you have on your smartphone?

- iOS
- Android
- Windows Phone
- Blackberry
- Others

**Question 8**

How much money would you invest into a medical app?

- Nothing
- Uo to 1 Euro
- Up to 5 Euro
- Up to 10 Euro
- More than 10 Euro

**Frage 9**

Are there situations during your daily work in which you wish to have support by a medical app?

(It is possible to select several answers)

- No, I do not need any more apps
- Yes, in the classification area
- Yes, in the imaging area
- Yes, in the treatment area
- Yes, in the education area

**Question 10**

How would you proceed if you diagnosed a musculoskeletal tumor

(It is possible to select several answers)

- I look into e reference book
- I inform myself on the internet
- I ask a colleague
- I refer the patient to a specialty center
- Usually I do not need any help
