# Supplementary material for: Apps in Clinical Practice: Usage Behaviour of Trauma Surgeons and Radiologists in Northern Germany
Source: Int J Telemed Appl. 2023 Aug 2;2023:3930820. doi: 10.1155/2023/3930820 (PMC10412380; doi:10.1155/2023/3930820)
Supplement: Supplementary 2 — App ranking of the trauma surgeons. [file 3930820.f2.docx]

App ranking trauma surgeons

| **App Name** | **Mentions** | **Category** |
| --- | --- | --- |
| AO Surgery Reference | 33 | Treatment |
| Arznei Aktuell | 33 | Medication |
| Amboss | 23 | Reference, Diagnostic Treatment |
| AO classification | 13 | Classification |
| Orthorad | 14 | Referece |
| Siilo | 7 | Communication |
| Orthobullets | 7 | Education |
| eref | 7 | Reference |
| pro Implant foundation | 6 | Treatment |
| MyAO | 5 | Communication |
| AO Trauma Orthogeriatrics | 5 | Treatment |
| Pedi help | 5 | Calculator |
| Embryotox | 4 | Medication |
| Arzneimittel Pocket | 4 | Medication |
| Corona warn App | 4 | Others |
| Multiplier | 4 | Calculator |
| Arthrex surgeon App | 4 | Treatment |
| Pedbone | 4 | Reference |
| Paley Growth | 3 | Calculator |
| Med Calc | 3 | Calculator |
| mRay | 3 | Imaging |
| ATLS | 3 | Treatment |
| Touch surgery | 3 | Reference |
| Trigger Points | 3 | Reference |
| FoBi App | 3 | Ärztekammer |
| Medscape | 3 | Reference |
| Neuromind | 2 | Treatment, Classification |
| Atlas Anatomie 3D | 2 | Reference |
| PubMed | 2 | Reference |
| Essential Anatomy | 2 | Reference |
| Orthoref | 2 | Classification |
| VuMedi | 2 | Education |
| UpToDate | 2 | Treatment |
| univadis | 1 | Education |
| AO surgery advice | 1 | Treatment |
| compendium | 1 | Reference |
| Herodikos | 1 | Treatment |
| STIKO | 1 | Reference |
| ESC guidelines | 1 | Reference |
| Defikataster | 1 | Others |
| Krankenhausapp zur internen Kommunikation | 1 | Communication |
| Doctolib | 1 | Communication |
| Skoliometer | 1 | Calculator |
| CobbMeter | 1 | Calculator |
| BDC mobile | 1 | Reference |
| antidoping | 1 | Treatment |
| Forta | 1 | Medication |
| AO spine | 1 |  |
| Palliative Care Tools | 1 | Treatment |
| guidelines | 1 | Reference |
| Bone Age | 1 | Calculator |
| iGOÄ | 1 | Gebührenordnung |
| GN Notfallfibel | 1 | Treatment |
| GisChem | 1 | Reference |
| MediCad | 1 | Planning Arthroplasty |
| Ehra | 1 | Reference |
| Whatsapp | 1 | Communication |
| Gelbe Liste | 1 | Medication |
| Ped(z) | 1 | Calculator |
| BosTT | 1 | Reference |
| Health | 1 | Others |
| Traumapedia | 1 | Reference |
| Phyto | 1 | Others |
| Notaufnahme App | 1 | Others |
| ICM Philly | 1 | Education |
| icd Auskunft | 1 | Others |
| Acetabular fractures | 1 | Classification |
| Spine Score | 1 | Calculator |
| DocCheck Flexicon | 1 | Reference |
| Aidminutes-rescue | 1 | Others |
| Visible body | 1 | Reference |
| athlete central | 1 | Anti-Doping |
| Antibiotika/Antiinfektiva | 1 | Treatment |
| Radiopedia | 1 | Reference |
| JBJS clinical classroom | 1 | Education |
| complete anatomy | 1 | Reference |
| Impf. Infos | 1 | Reference |

Summary:

| Treatment: | 12 |
| --- | --- |
| Reference: | 23 |
| Classification: | 3 |
| Calculator: | 9 |
| Education: | 5 |
| Medikation: | 5 |
