# Supplementary material for: Apps in Clinical Practice: Usage Behaviour of Trauma Surgeons and Radiologists in Northern Germany
Source: Int J Telemed Appl. 2023 Aug 2;2023:3930820. doi: 10.1155/2023/3930820 (PMC10412380; doi:10.1155/2023/3930820)
Supplement: Supplementary 3 — App ranking of the radiologists. [file 3930820.f3.docx]

App ranking radiologists

| **Name** | **Anzahl** | **Kategorie** |
| --- | --- | --- |
| MRI Essentials | 17 | Imaging |
| Imaios eAnatomy | 17 | Reference |
| eRef Thieme | 17 | Reference |
| Amboss | 13 | Reference, Diagnostic, Treatment |
| Arznei aktuell | 8 | Medication |
| mRay | 6 | Imaging |
| UpToDate | 6 | Reference |
| Radiology Assistant | 6 | Imaging |
| MeVis Recist 1.1 | 5 | Calculator |
| CMR guide/sequence | 5 | Reference |
| Pedz | 4 | Calculator |
| Fobi App | 4 | Ärztekammer |
| Nodule | 3 | Reference |
| Siilo | 3 | Communication |
| Horos Mobile | 3 | Imaging |
| Ärzteblatt | 3 | Journal |
| Flexikon | 4 | Reference |
| Radiopaedia | 3 | Imaging |
| Pedbone | 2 | Reference |
| Vascular variants | 2 | Reference |
| Corona Warn App | 2 | Sonstiges |
| StaDx | 2 | Sonstiges |
| CancerTNM | 2 | Calculator |
| MRI made easy | 2 | Imaging |
| AO surgery reference | 2 | Treatment |
| Leitlinienprogramm Onkologie | 1 | Reference |
| edx | 1 | Education |
| Brainomics | 1 | Others |
| Joyn | 1 | Others |
| Dose Calculator | 1 | Calculator |
| Rea Leitlinie | 1 | Reference |
| eGFR | 1 | Calculator |
| Rote Liste | 1 | Reference |
| Face2gene | 1 | Reference |
| Leitlinien DGIM | 1 | Reference |
| Planerio | 1 | Organizer |
| JiggleMed | 1 | Communication |
| iGOÄ | 1 | Gebührenordnung |
| GoToWebinar | 1 | Education |
| Osirix | 1 | Imaging |
| Researcher | 1 | Journal |
| aidminute.rescue | 1 | Others |
| readCubePapers | 1 | Others |
| Evernote | 1 | Organizer |
| Arzneimittel pocket | 1 | Medication |
| Miethke | 1 | Treatment |
| Bookshelf | 1 | Education |
| Diagnosia | 1 | Medication |
| Bisniak calculator | 1 | Calculator |
| Meditorium | 1 | Education |
| inkling books | 1 | Reference |
| MRI sequences | 1 | Imaging |
| RadiologiQ | 1 | Imaging |
| TI-Rads | 1 | Imaging |
| DZG | 1 | Reference |
| MDcalc | 1 | Calculator |
| CIRSE | 1 | Others |
| Berlin Case Viewer | 1 | Imaging |
| Clinical Key | 1 | Reference |
| ResearchGate | 1 | Reference |

Summary:

| Imaging: | 11 |
| --- | --- |
| Reference: | 17 |
| Medication: | 3 |
| Treatment: | 2 |
| Calculator | 7 |
| Communication | 2 |
| Education | 4 |
