# Supplementary material for: Apps in Clinical Practice: Usage Behaviour of Trauma Surgeons and Radiologists in Northern Germany
Source: Int J Telemed Appl. 2023 Aug 2;2023:3930820. doi: 10.1155/2023/3930820 (PMC10412380; doi:10.1155/2023/3930820)
Supplement: Supplementary 4 — Description of the most mentioned apps. [file 3930820.f4.docx]

Description of the most mentioned apps

AO surgery reference and AO classification:

These two apps are fracture management tools based on current clinical principles, practices, and available evidence, and a fracture classification reference tool, respectively. The apps were developed in 2010 in Davos, Switzerland by the AO Foundation - a medically-led non-profit organization, a global network of surgeons specializing in the treatment of trauma and musculoskeletal dysfunction. The apps have app store ratings of 5/5 and 3/5, respectively, are available for free, and are designed for trauma surgeons.

Arznei aktuell:

This app is a drug database with all relevant information on all prescription and pharmacy drugs in Germany, as well as all medical devices with medicinal character and OTX preparations. It was developed in Germany in 1013 by ifap Service - an institute for doctors and pharmacists GmbH, is available free of charge and has a rating of 3.4/5 in the App Store.

Amboss:

This app provides an interdisciplinary knowledge and reference tool for medical students and physicians on diagnostics, treatment, medication, and exam preparation. It provides answers to medical questions, comprehensive information on clinical pictures, as well as diagnostic and therapeutic options including guideline-based medication recommendations. The app is intended for everyday ward and practice life, clinical traineeship and PJ, all exam preparations and preparation for the specialist examination. Different modes can be selected: doctor, clinic, pre-clinic. Developed in Germany in 2012 by an international team of physicians, scientists and software developers, it is available for free and has a rating of 4.8/5 in the App Store.

Orthorad:

The app provides a reference (image) database of traumatic skeletal radiography. It was developed in 2013 by Christian Schramm in Germany and can be used by MTRAs to obtain information on storage and cassette format, among other things. Physicians, on the other hand, receive information on radiographic anatomy, reference data on the human skeleton, and tips on common traumatologic and orthopedic conditions. The app costs a one-time fee of €3.99 and has a rating of 4.9/5 in the App Store.

eRef Thieme:

Georg Thieme Verlag developed this app for physicians in 2014, which allows e-books and e-journals to be downloaded to smartphones and read offline. The app is free and rated 4.2/5.

MRI-Essentials:

Developed in 2017 by Wolfgang Fischer for physicians, this app is a reference and textbook on MRI of the musculoskeletal system. The basic version offers short text sections with sketches, while the Pro version offers detailed texts and over 4000 case studies. The basic version of the app is free, the Pro version costs 39,99€/year by subscription. The app has a rating of 3.9/5.

IMAIOS e-Anatomy:

This app is an atlas of human anatomy with > 15,000 images, including CT,- MRI and PET images, X-rays, angiograms, anat. Cross sections, diagrams and illustrations. Developed in 2008 by Imaios - a medical imaging and e-learning company - in the U.S. and France, the app is free and rated 4.8.
